# Pedagogic Approach in the Surgical Learning: The First Period of “Assistant Surgeon” May Improve the Learning Curve for Laparoscopic Robotic-Assisted Hysterectomy

**DOI:** 10.3389/fsurg.2016.00058

**Published:** 2016-11-02

**Authors:** Angeline Favre, Stephanie Huberlant, Marie Carbonnel, Julie Goetgheluck, Aurelie Revaux, Jean Marc Ayoubi

**Affiliations:** ^1^Obstetrics and Gynecology Department, Foch Hospital, Suresnes, France

**Keywords:** robotic-assisted hysterectomy, laparoscopy, learning curve, educational program

## Abstract

**Background:**

Hysterectomy is the most frequent surgery done with robotic assistance in the world and has been widely studied since its emergence. The surgical outcomes of the robotic hysterectomy are similar to those obtained with other minimally invasive hysterectomy techniques (laparoscopic and vaginal) and appear as a promising surgical technique in gynecology surgery. The aim of this study was to observe the learning curve of robot-assisted hysterectomy in a French surgical center, and was to evaluate the impact of the surgical mentoring.

**Methods:**

We retrospectively collected the data from the files of the robot-assisted hysterectomies with the Da Vinci^®^ Surgical System performed between March 2010 and June 2014 at the Foch hospital in Suresnes (France). We first studied the operative time according to the number of cases, independently of the surgeon to determine two periods: the initial learning phase (Phase 1) and the control of surgical skills phase (Phase 2). The phase was defined by mastering the basic surgical tasks. Secondarily, we compared these two periods for operative time, blood losses, body mass index (BMI), days of hospitalizations, and uterine weight. We, finally, studied the difference of the learning curve between an experimented surgeon (S1) who practiced first the robot-assisted hysterectomies and a less experimented surgeon (S2) who first assisted S1 and then operated on his own patients.

**Results:**

A total of 154 robot-assisted hysterectomies were analyzed. Twenty procedures were necessary to access to the control of surgical skills phase. There was a significant decrease of the operative time between the learning phase (156.8 min) compared to the control of surgical skills phase (125.8 min, *p* = 0.003). No difference between these two periods for blood losses, BMI, days of hospitalizations and uterine weight was demonstrated. The learning curve of S1 showed 20 procedures to master the robot-assisted hysterectomies with a significant decrease of the operative time, while the learning curve of S2 showed no improvement in operative time with respect to case number.

**Conclusion:**

Twenty robot-assisted hysterectomies are necessary to achieve control of surgical skills. The companionship to learn robotic surgery seems also promising, by improving the learning phase for this surgical technique.

## Introduction

Since the first hysterectomy with robotic assistance, the emergence of this new surgical technique was widely studied (1–5). In 2010, 30% of all hysterectomies were done laparoscopically, while 10% were done with robotic assistance (2). The robotic surgery represented by the Da Vinci^®^ System offers many advantages, such as improved ergonomics and tremor reduction. The system permits seven degrees of freedom and 360° pronosupination amplitude making stitches an easier task. The vision compared to conventional laparoscopy was improved by a 3D Camera (1–5).

Similar to Tapper et al. in 2014 (6), our previous results presented robotic surgery as safe and useful (7), further allowing us to enlarge the indication for robotic surgery. Its feasibility was demonstrated in functional and oncological surgeries (8). Bogani et al. (9) demonstrated that the implementation of robotic-assisted surgery for endometrial cancer staging improves patient outcomes as lower post-operative complication rate, lower blood transfusion rate, longer median operating time, shorter median length of stay, and lower readmission rate compared to patients undergoing open staging. Furthermore, Serati et al. (9) brought to light the feasibility of robot-assisted sacrocolpopexy. In a prospective study, including 94 hysterectomies, we compared the robotically assisted hysterectomy to vaginal hysterectomy for benign diseases (7). Our results showed a reduced blood loss, post-operative pain, and length of hospital stay but was associated with a longer operative time and higher cost. Even if the gynecological surgeries represent a low part of the robotic activity in France, hysterectomy is the most frequently performed intervention in robotic surgery in the world (10, 11).

The objective of this study was to evaluate the learning curve of surgical hysterectomy with robotic assistance. The learning curve is defined as the number of cases necessary to stabilize the operative time (12). This tool was largely used for supervision and control in surgical education (13). A systematic revue of the learning curve of robotic surgery made by Schreuder et al. concludes that the robotic surgical training consists of system training and procedural training (14). The system training should be formally organized and competence based, whereas the procedural training should be approached stepwise.

In comparison to laparoscopic hysterectomy, the learning curve is stiffer and the required case number leading to operation times of an experienced surgeon is lower (15, 16). The hypothesis of the importance of surgical mentoring in the surgical education led to compare the learning curve of two surgeons with different surgical experience. We further investigated the changes with respect to blood loss, uterine weights, and hospitalization duration for growing surgeon experience. The uterine weight was a parameter of clinical outcomes because it is a parameter to consider choosing the surgical approach. Silasi et al. (17) showed us that robotic surgeries for very large myomatous uteri are feasible and have minimal morbidity even in morbidly obese patients than laparotomy. So it seemed interesting to consider this parameter in our study.

## Materials and Methods

We retrospectively analyzed the data picked up in the medical files of the patients who underwent a robotic-assisted hysterectomy between March 2010 and June 2014 at the Foch hospital in Suresnes (France). The surgeries were performed with the Da Vinci^®^ Surgical System, available in our center since 2010.

The demographics parameters collected included age, parity, body mass index (BMI), hormonal status, medical history of abdominal surgery, operative indication, and the uterine weight. We also detailed whether the hysterectomy was associated with adnexectomy (non-conservative hysterectomy) or not (conservative hysterectomy).

We secondarily observed the operative time in minutes (elapsed time between skin incision and skin closure). For the first 60 interventions, we additionally analyzed three periods of time: the trocar time (between the skin incision and the insertion of the last trocar), the docking time (between the insertion of the last trocar and the beginning of robotic surgery), and the robot time (for the surgeon working on the console). We also analyzed the anesthesia duration, the blood loss (the difference between hemoglobin at day 1 compared to the hemoglobin before surgery), and surgical complications with a 3 months follow-up. A histological analysis of the uterus was realized for all cases.

We first studied the operative time according to the number of cases, independently of the experience of surgeons. This allowed us to determine two periods: the learning phase and the state of stabilization (control of surgical skills phase). The learning phase is defined as the period where the operation time was getting shorter and the learning plateau as the period where the operation time was similar procedure after procedure (16, 18). We compared these two phases using the operative time, blood losses, BMI, days of hospitalizations, and uterine weight.

We secondarily studied the difference of the learning curve between two surgeons. The first surgeon was an experimented surgeon and had performed over 100 laparoscopic hysterectomies. The second surgeon had less experience in laparoscopic surgery. He just finished his fellowship in gynecological surgery and had performed less than 20 laparoscopic hysterectomies.

The statistical analysis used was the Student *t*-test. The significance was set for *p* < 0.05. We graphically represented the operative time vs. case number for all hysterectomies. In order to define the two phases, we identified the case number for which the curve was getting to a level of stabilization and defined our learning phase as the first part of the curve, and the control of surgical skills phase when operative time reached its minimum. Finally, we compared the two periods for all hysterectomies, for surgeon 1 and surgeon 2. Results are presented with mean (±SD), number (*n*), and percentage (%).

## Results

Overall 154 robotic hysterectomies were realized between March 2010 and June 2014 at the Foch hospital in Suresnes (France). The average age of our patients was 48.1 (34.8–61.4) years and the average BMI was 25 (20.2–29.4) kg/m^2^. Thirty-nine percent of the patients were menopausal, 37% had a history of abdominal surgery, and 90% underwent surgery for a benign pathology. Characteristics of the population are detailed in Table 1.

**Table 1:** **Demographic data (SD)**.

	*n* (Percentage)	Average (±SD)
Age		48.1 (±13.3)
BMI		24.8 (±4.6)
Gestity/parity		1.8 (±1.9)/1.4 (±1.4)
Menopausal patients	39 (25.3)	
Hormone replacement therapy	15 (9.7)	
History of abdominal surgery	58 (37.7)	
Malign disease	20 (13.0)	
Benign disease	133 (90.3)	

The surgical and post-operative characteristics are detailed in Table 2. All hysterectomies were total hysterectomies. The complementary interventions were 11 adhesiolysis (6.6%), 2 bilateral salpingectomies (1.2%), 2 adnexectomies (1.2%) and 2 ovarian cystectomies (1.2%).

**Table 2:** **Surgical and post-operative data**.

	**Average (±SD)**
Uterine weight	158.9 (±147.1)
Blood loss	62.7 (±97.1)
Hemoglobin difference	1.2 (±1.0)
Operation time	129.7 (±44.6)
Anesthesia time	189.5 (±51.4)
Day of hospitalization	3.5 (±1.8)
	***n* (Percentage)**
Conservative hysterectomies	47 (30.5)
Non-conservative hysterectomies	106 (68.8)
Transfusion	1 (0.6)
Laparoconversion	0 (0)
Complimentary interventions	18 (11.7)
Complications	19 (12.3)
Pre-operative complications	7 (4.5)
Post-operative complications	12 (7.8)

There was no conversion to open procedure in our study. The complication rate was 12.3% (19/154). The pre-operative complications were two bladder injuries (1.2%), two bowel injuries (1.2%), one pre-operative hemorrhage (0.6%), one bladder and bowel injury (0.6%), and one arm compression leading to post-operative pain (0.6%). The incidence of post-operative complication was 7.8%: 2 vaginal cuff dehiscences (1.2%), 4 hematomas (2.4%), 2 surgical site infections (1.2%), 1 patient who experienced a transitory blurred vision (0.6%), 1 vaginal collection (0.6%) were observed. One patient suffered secondarily from stress urinary incontinence solved in 3 weeks and one patient had a pulmonary embolism and a peritonitis with drained peritoneal abscesses (0.6%). Three (1.8%) of these patients were operated again because of complications of the primary surgery.

**Figure 1:**
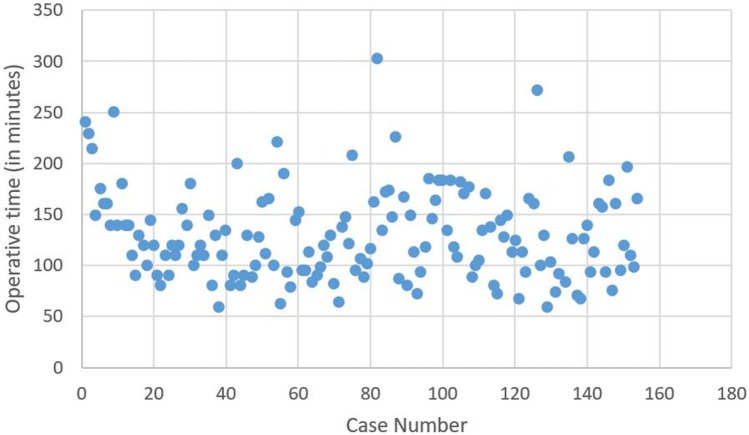
**Operative time (in minutes) depending on case number, independent of the surgeon**.

The learning curve, based on total operative time, without distinguishing any surgeon (Figure 1) for the 154 robotic hysterectomies shows two periods: the first learning period for the 20 first cases and the control of surgical skills phase for the 134 other cases.

There is significant decrease of the operative time between the 1st and the 20th surgery (first period) with a mean of 156.8 min compared to the second phase corresponding to the following surgeries, with a mean of 125.8 min (second period) (*p* = 0.003). No significant difference for operative time was shown in the control of surgical skills phase between the 21st and the 154th case.

We also observed a significant difference between the two periods for the anesthesia duration (228 vs. 184 min, *p* < 0.01), the trocar time (11 vs. 8 min, *p* = 0.01), the docking time (9.2 vs. 5.6 min, *p* < 0.01) and the robot time (120 vs. 77 min, *p* < 0.01).

There is no difference for these two periods concerning age, BMI, uterine weight, or blood loss (Table 3). Even if no significant difference was shown between these two periods concerning operative complications, there was no complication in the first period.

**Table 3:** **Comparison between the average data for the first 20 cases and the following ones**.

	Average of the first 20 cases	Average of subsequent cases	*p*-Value
Age	48.7	47.9	ns
BMI	24.8	24.8	ns
Uterine weight	176.8	139.0	ns
History of abdominal surgery	0.3	0.4	ns
Blood loss	43.2	65.8	ns
Operative time	156.8	125.8	0.003
Anesthesia time	228.5	183.6	<0.01
Trocar time	11.0	8.4	0.01
Docking time	9.2	5.6	0.002
Robot time	120.3	77.4	<0.01

The learning curve has secondarily been studied for 2 surgeons who practiced 81 and 46 robotic hysterectomies among the 154 procedures, respectively.

**Figure 2:**
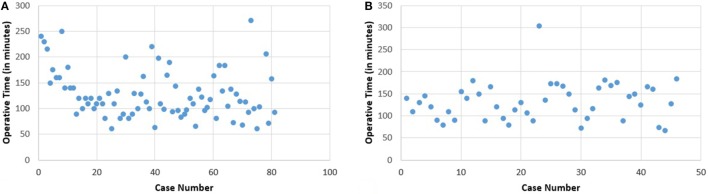
**Operative time (in minutes) vs. case number for S1 (A) and S2 (B)**.

The first surgeon, named “S1” who practiced 81 robotic hysterectomies, has a learning curve showing the need of 20 operations to stabilize the operative time (Figure 2A). There is no significant difference in the control of surgical skills phase for the duration of the surgical procedures.

There is a significant difference between the mean operation time for the first 20 cases (152.5 min) and the subsequent (119.6) (*p* = 0.006). There is no difference in these two groups concerning age, BMI, uterine weight, history of abdominal surgery, and blood loss (Table 4).

**Table 4:** **Comparison between the average of the data for the first 20 cases and the following ones, for “S1” and “S2”**.

Surgeon	S1			S2		
	Average of the first 20 cases	Average of subsequent cases	*p*-Value	Average of the first 20 cases	Average of subsequent cases	*p*-Value
Age	46.6	46.2	ns	49.0	52.8	ns
BMI	24.9	24.2	ns	25.5	26.1	ns
Uterine weight	147.6	155.5	ns	169.5	171.6	ns
Previous surgery	0.4	0.3	ns	0.3	0.6	ns
Blood loss	48.9	71.0	ns	33.0	68.4	ns
Operative time	152.5	119.6	**0.006**	121.5	141.2	ns
Anesthesia time	222.0	180.8	**0.005**	184.2	197.8	ns
Trocar time	11.9	7.7	**0.0002**	Not available	Not available	
Docking time	10.0	5.0	**0.0003**	Not available	Not available	
Robot time	121.8	72.0	**0.0003**	Not available	Not available	

*Bold font was chosen to highlight the statistical significance*.

The second surgeon’s (named “S2”) learning curve shows no improvement in operative time with respect to case number. There is no evolution noticed either for BMI, uterine weight, or blood loss (Figure 2B; Table 4).

No complications occurred for the first surgeon’s hysterectomies. The second surgeon experienced four pre-operative and eight post-operative complications. All of them were in the control of surgical skills phase. There is no significant difference between the first and the second surgeon for the complication rate.

## Discussion

This study was realized in order to determine the learning curve in robotic surgery especially for the practice of hysterectomy, in a French center. We focus on the difference between two surgeons separate by their surgical experience. Distinguishing surgeon 1 and surgeon 2 allowed us to study the impact permitted of the surgical mentoring and its benefits for the learning of new techniques.

Surgeon 1 was the most experienced surgeon. He had practiced several laparoscopic hysterectomies and was the first practicing on the Da Vinci^®^ surgical system and discovering it. Surgeon 2 had done only 20 hysterectomies by laparoscopy. He assisted surgeon 1 from the very beginning of the robotic surgery in our center and took more and more responsibilities in the 15 first hysterectomies before doing his own surgeries. It seems to be very interesting studying the effect of the role of “assisted surgeon” in the pedagogic approach of the surgical education (19).

The learning curve of this study shows the necessity of 20 cases to master the robotic surgery technique. The operative times are then significantly different between the learning phase and the control of surgical skills phase. The two groups (the 20 first cases and the following ones) are similar in terms of BMI, uterus weight, age, history of abdominal surgery, and blood loss.

The same learning curve is brought to light for the experimented surgeon (S1), such as seen in the literature (18, 20). By contrast, the learning curve for the less experimented surgeon (S2) was different from the first one, and we did not observe a cut off at 20 cases. As he assisted surgeon 1 in 15 interventions first before operating on his own patients, it suggests that he acquired his experience near to surgeon 1. It seems that the learning of the robotic surgery technique can be improved by assisting another surgeon in a fellowship. We so avoid the learning phase by doing the apprenticeship with an experimented fellow. Crane et al. showed that training program with robot-assisted is possible and does not decrease the robotic operative efficiency (19). Our results go in the same direction.

The demographics of our population were similar to the literature as it was reported by Albright et al. (21). This meta-analysis only reported one count lower of patient with previous abdominal surgery (40–90% for Albright et al.) that it could explain the difference in terms of complication that those described in our cohort (21). The complication rate observed in this study (12.3%) is similar to the rate observed in recent prospective studies (16). Only two patients (1.2%) were concerned by vaginal cuff dehiscence. In a recent Corean study, Kim et al. evaluated the risk factors for vaginal cuff dehiscence after 604 hysterectomies (22). They found out that total laparoscopic hysterectomies were more at risk of dehiscence (5.68%) and that it occurs more often when the vaginal suture was done by vaginal way. No laparoconversion was necessary in our study. In the literature, the rate of it is 0–2% (11, 23).

We choose to study the learning curve of our experimented surgeon because we found it interesting to know how much robotic surgeries were necessary to master the robotic hysterectomy for a surgeon who is totally aware of the technic for the laparoscopic hysterectomy. We also found it interesting to show that companionship is a good way to learn robotic surgery as for surgeon 2. The originality of our work is to bring to light these two aspects of the apprenticeship of the same new surgical technique: the first surgeon by “doing it yourself” and the second surgeon with learning from a master.

The global learning curve based on the operative time, independent of the surgeon, is similar to the ones in the literature (20). Lin et al. studied the learning curve for a single surgeon who has practiced 100 robotic hysterectomies (18). After a multivariable modeling with linear regression, they show that there is a flattening of the learning curve after 20–30 hysterectomies, which is similar to our experience for surgeon 1. A multivariate analysis was not possible in our study because the case number was too small regarding the necessary power to obtain significant results. The heterogeneity of surgical procedure and the difference between the surgical indications limited the obtention of group with sufficient number of patients. Geller et al. compared the 20 first sacrocolpopexy and concomitant hysterectomies to the 127 next (20). They demonstrated an improvement of efficiency for all operative steps with greatest difference in intracorporeal suturing and overall operative time also after 20 procedures as our study. Even though it is not the same surgery, it suggests that 20 is the cut off for the learning curve of other benign procedures. Results did not show differences on the learning curve between experimented and fellows (24, 25). In the study from Sandadi et al., the fellows improved their operative time from 60 min for a robotic hysterectomy in 2009 to 31 min 2 years later with same indications that in our study (24). This corresponds to 33 cases what is different from our series. The cases necessary to master this surgery for fellows seems to be more important than for experimented surgeons as suggested in our study. It could be explained because of the similar techniques for hysterectomies in laparoscopic and robotic surgery.

Even if our study suggests that experimented surgeons realize a better handling with better dexterity than fellows, it is interesting to evaluate the impact of robotic surgery on a surgical program. The S2 learned the docking procedure by helping S1. This is why the learning curve for S2 is flatter. Soliman et al. proposed to integrate the robotic surgery to the learning program for fellows in oncologic gynecologic surgery (25). The interventions done by the fellows significantly increased in 3 years with console times and lymph node yields similar to faculty surgeons. The learning curve for laparoscopic hysterectomy requires more cases before reaching the learning plateau than for robotic surgery. For Hwang et al., the learning curve for laparoscopic hysterectomies is 40 cases (26). Eltabbakh et al. show in a retrospective study that the performance for one surgeon increases after 25 and 50 cases for laparoscopic hysterectomies in endometrial cancer (27). These learning curves are an interesting way to observe and evaluate the mentoring in robotic surgery, by comparing the results before and beyond 20 procedures needed to obtain a learning plateau.

Our study has some limitations that need to be detailed. First the retrospective analysis limited the methodology, even if the number of case was important to the evaluation, compared to the literature (18, 26). Indeed, the number of surgical procedures was higher than in most part of series described in the literature about the learning curve in surgical practice, especially for laparoscopic hysterectomy with robotic assistance (18, 26, 27).

Second, we include hysterectomies for benign and malign causes. In the case of malign diseases, the treatment consisted only in a hysterectomy. There was no lymphadenectomy or other complementary resection in our malign group. But we included patients with conservative and non-conservative hysterectomy what it could change the operative time. So it could be interesting to observe the learning curve about the same surgical procedure.

Third, the follow-up of our patients was only to 3 months and limited the collection of long-term complications.

## Conclusion

Despite its expensive investment, the robotic assistance for hysterectomy shows good performance and constitutes an interesting tool to optimize patient care. At long-term, the loss/profit ratio should be balancing to profit considering the costs of long time hospitalization and work stoppage. These learning curves are an interesting way to observe and evaluate the mentoring in robotic surgery, by comparing the results before and beyond 20 procedures needed to obtain a learning plateau. Our study stays up to date in order to evaluate our practices with the objective of increasing the protection of the patients and the quality of care. The present study presents a learning curve comparable to those described in the literature, with 20 interventions to master the robotic hysterectomy with a significant decrease of operative time. A prospective trial, conducted for the same surgical procedure, could improve the precision of this learning curve. Moreover, the observational time and the surgical mentoring before the first robotic handling could optimize the learning curve.

## Ethics Statement

The study received the approval of scientific committee of Foch Hospital.

## Author Contributions

AF contributed to the extraction of the data and writing the manuscript. SH writing the manuscript. MC, AR, and JG contributed to the revision of the manuscript. JMA contributed to the draft and the revision of the manuscript.

## Conflict of Interest Statement

The authors declare that the research was conducted in the absence of any commercial or financial relationships that could be construed as a potential conflict of interest.
